# Excess all-cause mortality in the USA and Europe during the COVID-19 pandemic, 2020 and 2021

**DOI:** 10.1038/s41598-022-21844-7

**Published:** 2022-11-03

**Authors:** Lauren M. Rossen, Sarah K. Nørgaard, Paul D. Sutton, Tyra G. Krause, Farida B. Ahmad, Lasse S. Vestergaard, Kåre Mølbak, Robert N. Anderson, Jens Nielsen

**Affiliations:** 1 National Center for Health Statistics, Centers for Disease Control and Prevention, 3311 Toledo Road, Hyattsville, MD 20782 USA; 2 Infectious Disease Epidemiology and Prevention, Statens Serum Institut, Copenhagen, Denmark; 3 Department of Veterinary and Animal Sciences, Faculty of Health and Medical Sciences, University of Copenhagen, Copenhagen, Denmark

**Keywords:** Infectious diseases, Epidemiology

## Abstract

Both the USA and Europe experienced substantial excess mortality in 2020 and 2021 related to the COVID-19 pandemic. Methods used to estimate excess mortality vary, making comparisons difficult. This retrospective observational study included data on deaths from all causes occurring in the USA and 25 European countries or subnational areas participating in the network for European monitoring of excess mortality for public health action (EuroMOMO). We applied the EuroMOMO algorithm to estimate excess all-cause mortality in the USA and Europe during the first two years of the COVID-19 pandemic, 2020–2021, and compared excess mortality by age group and time periods reflecting three primary waves. During 2020–2021, the USA experienced 154.5 (95% Uncertainty Interval [UI]: 154.2–154.9) cumulative age-standardized excess all-cause deaths per 100,000 person years, compared with 110.4 (95% UI: 109.9–111.0) for the European countries. Excess all-cause mortality in the USA was higher than in Europe for nearly all age groups, with an additional 44.1 excess deaths per 100,000 person years overall from 2020–2021. If the USA had experienced an excess mortality rate similar to Europe, there would have been approximately 391 thousand (36%) fewer excess deaths in the USA.

## Introduction

Since early 2020, both the USA and Europe have experienced excess mortality due, in large part, to COVID-19, a disease caused by SARS-CoV-2^1–5^. By April 14, 2022, there were 978,545 and 1,961,790 laboratory confirmed deaths from COVID-19 reported in the USA and the whole of Europe^6^, respectively.

Death counts from COVID-19 may be underestimated due to variability in testing access/utilization, non-random testing, lack of consistent reporting on death certificates, or misclassification of cause of death. Analyses of excess deaths from all-causes can overcome these challenges and estimate the full impact of the COVID-19 pandemic on mortality^7^. Excess deaths, defined as the difference between the observed number of deaths and the expected number for a given place and time period, also provide a consistent and comparable metric across countries, independent of baseline levels of mortality^8,9^. However, the methods or models used to estimate excess mortality often vary, making comparisons between regions using different methods difficult.

Since 2009, the network for European monitoring of excess mortality for public health action (EuroMOMO) has monitored weekly excess all-cause mortality in a large number of European countries^10^. EuroMOMO uses an established methodology for estimating excess mortality, calculated as the number of observed deaths minus the baseline expected number, that has been relied upon for more than a decade for the surveillance of influenza and other public health outcomes with an impact on mortality^11^.

The aim of this study was to apply the EuroMOMO methodology to estimate excess mortality in the USA and Europe during the COVID-19 pandemic, and to compare excess mortality by age group between the USA and Europe in 2020 and 2021.

## Results

We used data from the US National Vital Statistics System, which provides weekly provisional counts of deaths from all causes occurring in the USA from 2015 through 2021, by week of death, age group, and reporting jurisdiction (50 states, the District of Columbia [DC], and New York City)^12^. We also used data from the EuroMOMO hub at Statens Serum Institut, Denmark, which pools^11^ aggregated data collected from 25 European countries or subnational areas^13^ to provide weekly expected numbers of deaths and excess numbers of deaths, from all causes in Europe^13^.

We applied the EuroMOMO statistical model, which fits over-dispersed Poisson time series (with weekly data based on International Organization for Standardization [ISO] weeks) regression models to predict the expected number of deaths using data from the five preceding years^11,14^. To account for differences in population sizes between the USA and Europe, we estimated mortality incidence rates (deaths per 100,000 person years) for the expected baseline numbers of deaths, observed numbers of deaths and excess numbers of deaths.

Through 2020–2021, the USA experienced 6.9 million deaths while the estimated expected number of deaths was 5.8 million for a population of 329 million. Over the same period, the 25 European EuroMOMO countries or subnational areas, representing a population of 368 million, experienced 8.1 million deaths, while the expected number of deaths was 7.3 million. Together, these estimates correspond to 1.1 and 0.7 million excess deaths in the USA and the EuroMOMO countries or subnational areas, respectively, over the course of the pandemic.

### Comparisons

To account for differences in the age structure of the two populations, we age-standardized the mortality incidence rates using the direct method based on the joint weekly age distribution between the USA and the EuroMOMO countries or subnational areas throughout the study period. This age-standardization provides estimates of excess mortality incidence rates under the assumption that the age distribution is the same in the USA and Europe across the study period. We did not adjust for other factors such as sex, income, or area-level socioeconomic indicators.

We calculated rate differences in excess mortality incidence rates between the USA and Europe by subtracting rates for Europe from rates for the USA. Comparisons were made across the whole period (2020-W01 to 2021-W52) and for three time periods reflecting the three primary waves in excess mortality (wave 1: 2020-W01 to 2020-W39, wave 2: 2020-W40 to 2021-W26 and wave 3: 2021-W27 to 2021-W52).

Estimates of uncertainty around the rates or numbers of deaths are shown as 95% uncertainty intervals (± 1.96 standard deviations).

#### Excess mortality trends

**Figure 1:**
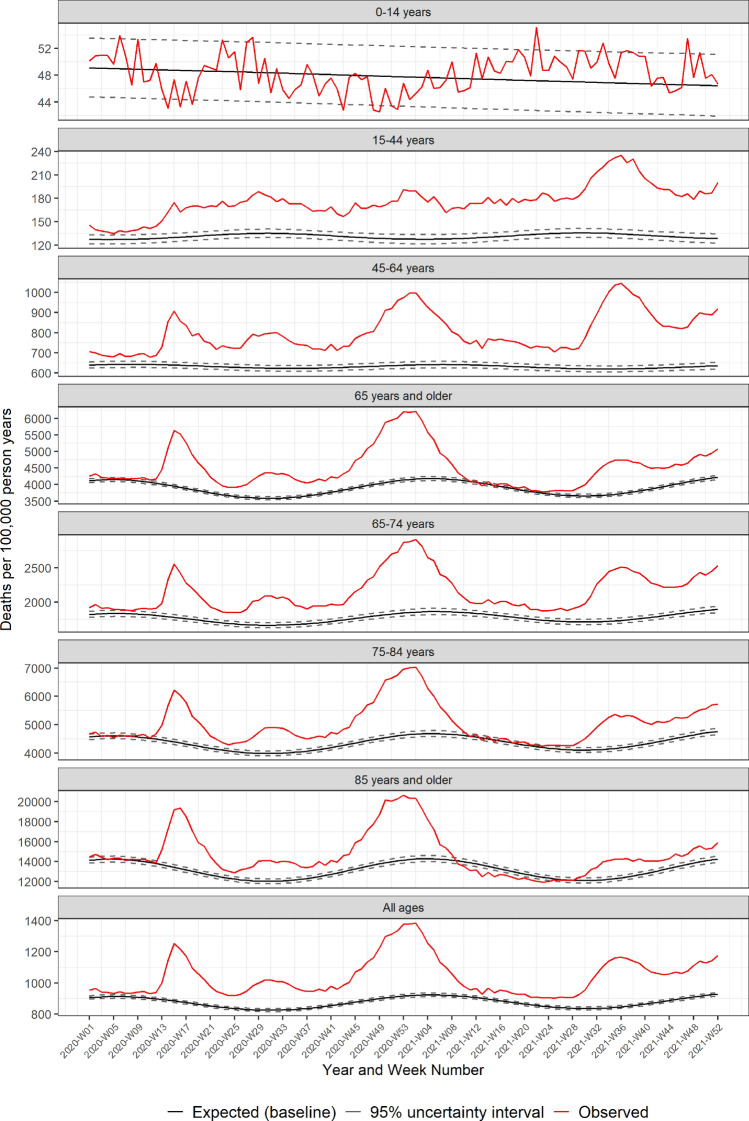
Pooled weekly expected and observed all-cause mortality rates (deaths per 100,000 person years), from 2020–2021: USA.

**Figure 2:**
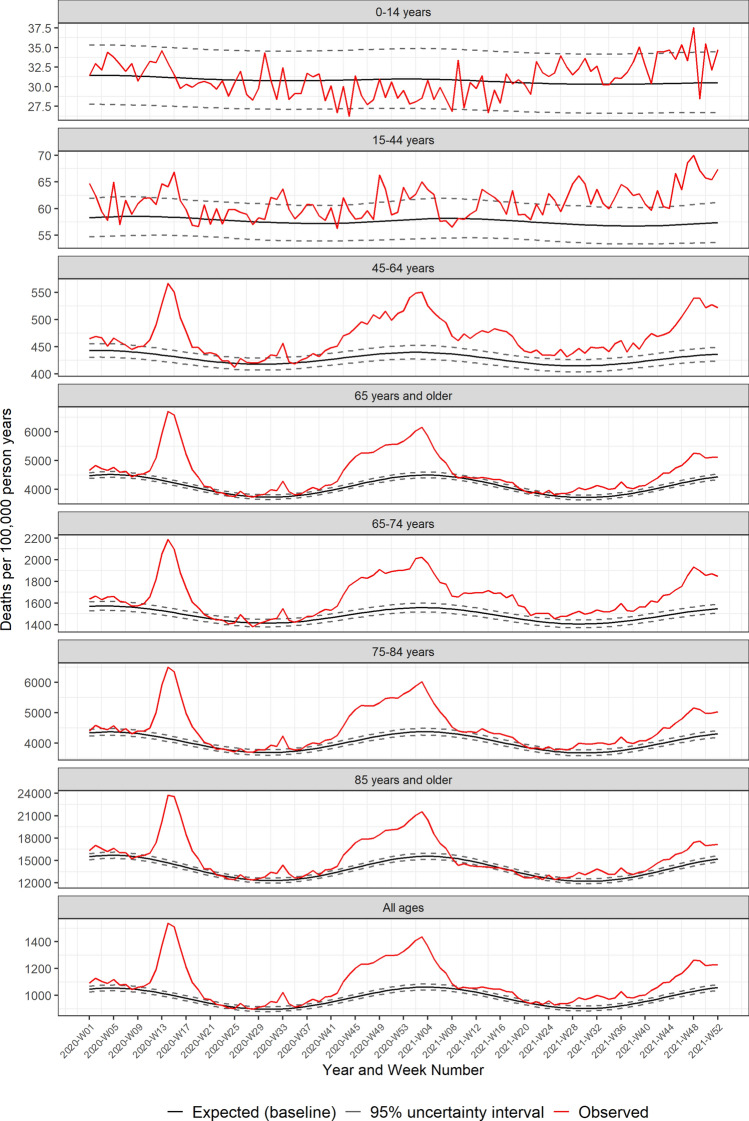
Pooled weekly expected and observed all-cause mortality rates (deaths per 100,000 person years), from 2020–2021: Europe.

**Figure 3:**
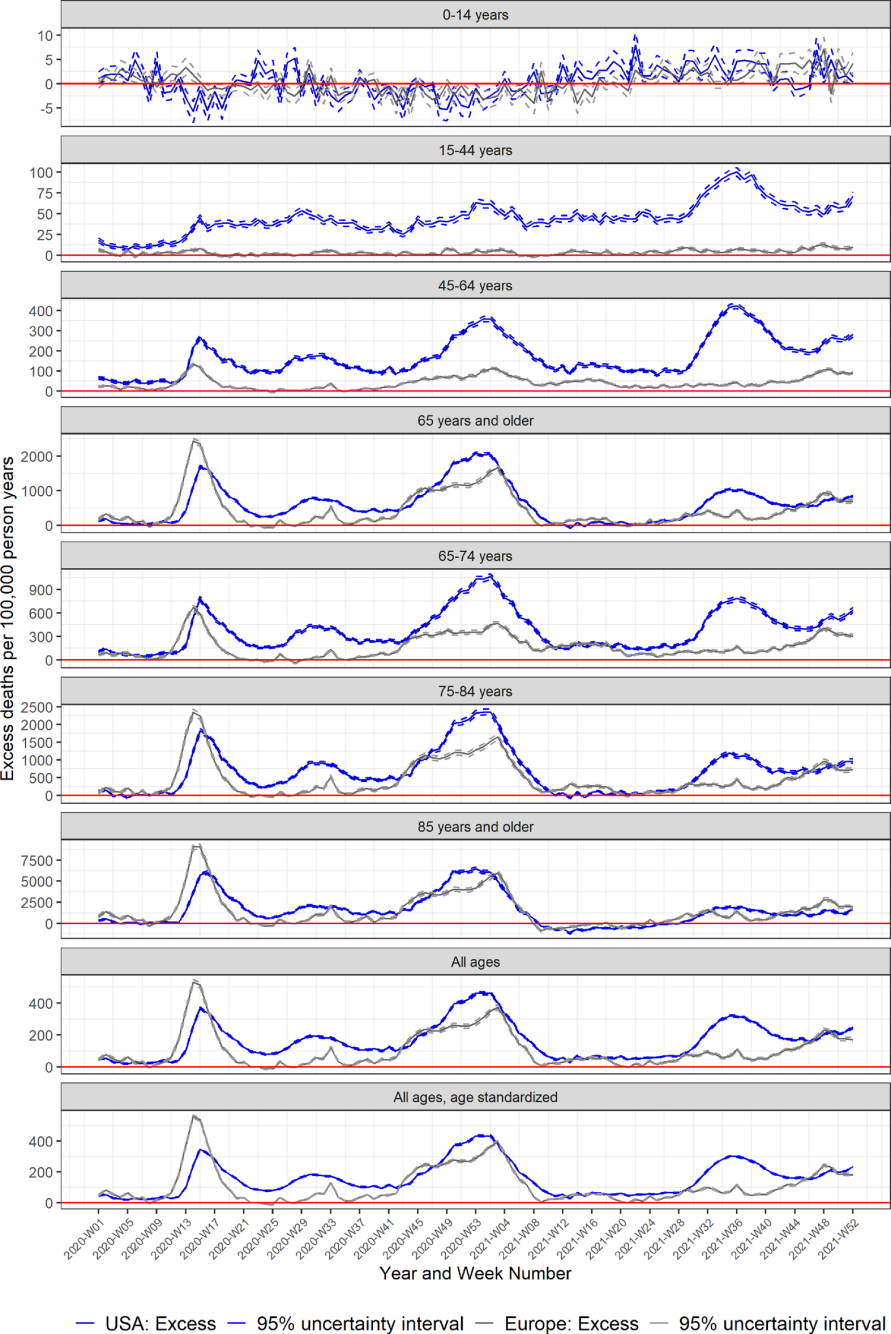
Pooled all-cause excess mortality rates (excess deaths per 100,000 person years), from 2020–2021: USA and Europe.

Both the USA and Europe experienced three main waves in excess mortality through 2020 and 2021 (Figs. 1 and 2). The initial wave began in late February (2020-W08) in Europe, and around two weeks later in the USA (Fig. 3). The European peak was initially higher than the USA, but excess mortality in Europe normalized by the end of May (2020-W22) with a minor peak in August (2020-W33), while mortality remained elevated in the USA over the summer of 2020, with a second smaller peak in July (2020-W31). The second large wave in both Europe and the USA started in autumn of 2020 and lasted through March 2021 (2021-W12). Thereafter mortality normalized in both the USA and Europe through mid-2021. The third wave began by mid-summer 2021 (2021-W27) in the USA, while Europe exhibited an increasing mortality through the latter half of 2021. By the end of 2021, mortality remained elevated in both the USA and Europe.

During 2020–2021, the USA experienced a cumulative excess mortality incidence rate of 154.5 (95% UI: 154.2–154.9) excess deaths per 100,000 person years across all age groups (Table 1), highest among those 65 years or older, 495.6 (491.4–499.7). Over the same period, Europe experienced an excess death incidence rate of 110.4 (109.9–111.0) across all age groups, also with higher rates among those 65 years or older, 378.0 (371.7–384.3). Overall, excess mortality incidence rates were higher in the USA than Europe for every age group, except for the youngest (0–14 years), with the largest rate differences observed among those 65–74 and 75–84 years. Accounting for the different age distributions across the two populations, there were an additional 44.1 (43.2–45.0) excess deaths per 100,000 person years in the USA than in Europe (Table 1).

**Table 1:** Pooled USA and European excess mortality rates (excess deaths per 100,000 person years) and rate differences, by time period and age group.

	2020 and 2021 2020-W01 to 2021-W52			2020-W01 to 2020-W39			2020-W40 to 2021-W26			2021-W27 to 2021-W52		
Age group	USA^1^	Europe^2^	Rate Difference	USA^1^	Europe^2^	Rate Difference	USA^1^	Europe^2^	Rate Difference	USA^1^	Europe^2^	Rate Difference
	Excess all-cause deaths per 100,000 person years (95% uncertainty interval)											
0–14 years	0.5 (0.4; 0.6)	0.3 (0.2; 0.3)	0.3 (− 0 .1; 0.7)	− 0.1 (− 0.2; 0.0)	0.2 (0.1; 0.3)	− 0.3 (− 0.9; 0.3)	− 0.1 (− 0.2; 0.0)	− 1.1 (− 1.3; − 0.9)	1.1 (0.4; 1.7)	2.4 (2.1; 2.8)	2.5 (2.2; 2.8)	0.0 (− 0.8; 0.7)
15–44 years	45.0 (44.6; 45.4)	3.6 (3.5; 3.7)	41.4 (40.9; 41.8)	30.7 (30.2; 31.3)	2.4 (2.2; 2.6)	28.3 (27.7; 29.0)	43.9 (43.3; 44.5)	2.7 (2.5; 2.9)	41.2 (40.6; 41.9)	67.9 (67.0; 68.8)	6.8 (6.5; 7.2)	61.1 (60.2; 62.0)
45–64 years	171.2 (170.3; 172.2)	40.3 (39.8; 40.9)	130.9 (129.7; 132.1)	113.5 (112.2; 114.9)	23.7 (23.0; 24.4)	89.8 (87.8; 91.8)	170.2 (168.7; 171.8)	50.2 (49.3; 51.1)	120.0 (118.0; 122.0)	259.5 (257.2; 261.7)	50.1 (49.0; 51.2)	209.4 (206.9; 211.9)
≥ 65 years	607.0 (604.2; 609.8)	452.1 (447.9; 456.2)	154.9 (148.2; 161.6)	495.5 (491.4; 499.7)	378.0 (371.7; 384.3)	117.6 (106.7; 128.4)	675.1 (670.4; 679.9)	538.1 (531.0; 545.4)	137.0 (125.9; 148.1)	668.4 (662.6; 674.3)	429.8 (421.7; 437.9)	238.7 (225.3; 252.0)
65–74 years	381.9 (379.5; 384.3)	166.6 (164.8; 168.3)	215.4 (211.8; 219.0)	258.5 (255.2; 261.8)	101.9 (99.5; 104.4)	156.5 (150.8; 162.3)	419.9 (415.9; 424.0)	219.8 (216.6; 222.9)	200.1 (194.2; 206.1)	507.5 (502.2; 512.9)	180.2 (176.6; 183.8)	327.4 (320.1; 334.6)
75–84 years	672.2 (667.4; 677.0)	455.9 (451.1; 460.6)	216.3 (207.5; 225.2)	522.4 (515.4; 529.5)	351.8 (344.8; 358.9)	170.6 (156.5; 184.8)	761.2 (753.0; 769.4)	574.3 (565.9; 582.8)	186.8 (172.2; 201.5)	758.8 (748.7; 769.0)	429.5 (420.2; 438.8)	329.3 (311.5; 347.1)
≥ 85 years	1522.3 (1509.6; 1535.1)	1450.3 (1433.7; 1466.9)	72.0 (42.8; 101.3)	1568.2 (1547.7; 1588.9)	1428.3 (1401.4; 1455.4)	139.9 (92.5; 187.3)	1688.6 (1666.9; 1710.4)	1573.1 (1545.1; 1601.2)	115.5 (66.9; 164.1)	1197.7 (1174.0; 1221.6)	1296.1 (1264.4; 1328.0)	− 98.4 (− 156.3; − 40.5)
All ages	164.7 (164.1; 165.3)	105.4 (104.5; 106.3)	59.3 (57.8; 60.7)	124.9 (124.1; 125.8)	84.5 (83.2; 85.9)	40.4 (38.1; 42.7)	176.0 (175.0; 177.0)	125.7 (124.1; 127.3)	50.3 (47.9; 52.7)	207.0 (205.7; 208.3)	105.6 (103.7; 107.4)	101.4 (98.5; 104.3)
All ages, age standardized^3^	154.5 (154.2; 154.9)	110.4 (109.9; 111.0)	44.1 (43.2; 45.0)	117.3 (116.8; 117.9)	88.6 (87.7; 89.5)	28.7 (27.2; 30.2)	159.2 (158.6; 159.7)	107.7 (106.9; 108.6)	51.5 (50.2; 52.8)	194.2 (193.4; 195.0)	110.7 (109.6; 111.9)	83.4 (81.6; 85.3)

^1^Fifty states and the District of Columbia.

^2^Twenty-five European countries or subnational areas in the EuroMOMO network.

^3^According to the joint USA and Europe age distribution.

#### Wave 1

During the first main wave of excess mortality occurring in spring and summer 2020 (2020-W01 to 2020-W39) the USA experienced an age-standardized excess incidence rate of 117.3 (95% UI: 116.8–117.9) (Table 1), with highest rates among those 65 years or older, 495.5 (491.4–499.7). Over the same period, Europe experienced age-standardized excess mortality incidence rates of 88.6 (87.7–89.5), also with higher rates among those 65 years or older, 378.0 (371.7–384.3). Excess mortality incidence rates were higher in the USA than Europe for every age group except persons aged 0–14 years, with the largest rate differences seen among those aged 75–84 years.

#### Wave 2

During the second wave in excess mortality (2020-W40 to 2021-W26), excess mortality was highest among older adults in both the USA and Europe. The age-standardized excess mortality incidence rates for the USA and Europe being 176.0 (175.0–177.0) and 125.7 (124.1–127.3), respectively.

#### Wave 3

From mid-summer 2021 to end of 2021 (2021-W27 to 2021-W52), the age-standardized excess mortality incidence rates were 207.0 (205.7–208.3) and 105.6 (103.7–107.4), in the USA and Europe, respectively. Over this period, excess mortality incidence rates were higher in the USA than in Europe for all age groups except those under 14 and those 85 and older. For this oldest age groups, excess mortality incidence rates were 98.4 (40.5–156.3) higher in Europe than in the USA during this time period.

## Discussion

The COVID-19 pandemic caused excess mortality in both the USA and Europe, with peaks in all-cause excess mortality in the spring 2020, over the winter 2020–2021 and at the end of 2021. Generally, the European countries included in EuroMOMO, mainly western-European countries, followed the pooled European patterns in excess mortality, though levels of excess mortality varied widely by country^10^. Patterns in the USA similarly varied by jurisdiction, with different jurisdictions exhibiting from two to five distinct peaks in excess deaths of varying magnitude^12^.

Between the waves, mortality normalized for all age groups in Europe (Fig. 2), while in the USA, elevated mortality was sustained among people 15–64 years of age (Fig. 1). The varying temporal patterns in excess mortality between the USA and Europe may be due to different mitigation and prevention strategies, vaccination coverage, and the predominance of different SARS-CoV-2 variants over time.

Our results for the USA are similar to estimates from Wang et al.^15^, who reported the total global number of excess deaths for 2020–2021, as well as estimates for many countries. In that study, Wang et al.^15^ estimated an excess of 1.1 million deaths in the USA, consistent with our findings. However, for the 25 EuroMOMO countries or subnational areas, our results show an estimated 0.8 million excess deaths, while Wang et al. estimated 1.0 million excess deaths. The estimates produced by Wang et al.^15^ have been criticized for being too large for some countries^16^, which would downwardly bias a differences between the USA and Europe.

Other studies have also found that the absolute number of excess deaths was highest in the USA during the first part of the pandemic, and that excess deaths among those 15–64 years were disproportionately high in the US relative to other countries^1,2,17–19^. Among younger age groups (except for children less than 15 years of age), excess mortality incidence rates were consistently higher in the USA than in Europe throughout 2020 and 2021. By contrast, among the oldest adults, excess mortality was initially higher in Europe during the first wave and toward the end of 2021. Factors contributing to these patterns and differences by age group between the USA and Europe might include population-level differences in sociodemographic, occupational, or health-related factors, social determinants, healthcare system differences, along with the percent of the older adult population residing in nursing homes or other residential facilities, and the potential differential impact of COVID-19 prevention, mitigation, and vaccination policies by age and geography^20–23^. Supplementary Figs. S1–S14 show the age-specific patterns in excess mortality incidence rates by region, with various temporal markers related to vaccine availability and other factors noted.

Several studies have identified higher baseline mortality rates in the USA relative to peer nations prior to and during the pandemic^24–27^. Consistent with these findings, Supplementary Fig. S15 illustrates the trends in mortality rates in the USA and Europe from 2017 to 2021, by age group. Prior research has reported that in 2017, the USA would have seen approximately 400,000 fewer deaths if the age- and sex-specific mortality rates matched those of five large European countries; a gap that has widened substantially since 2000^27^. Another recent study found that if the USA had the same age-specific mortality rates as other wealthy nations in 2019, there would have been 626,353 fewer deaths, with over half of these deaths occurring in people under 65 years of age^24^. These differences have grown during the pandemic^24,26^, with one recent analysis finding that approximately 1.1 million deaths in the USA, including half of deaths in people under 65 years, could have been averted in 2021 had age-specific mortality rates matched those of other wealthy countries^24^. The drivers of these long-standing differences in baseline mortality rates between the USA and other wealthy countries are complex, but include factors such as increasing drug overdose and suicide mortality as well as a plateau in heart disease mortality among other causes of death, with widening educational inequalities in mortality seen in the USA over the past several decades relative to other peer nations^25^.

In both the USA and Europe, the majority of excess mortality through 2020 and 2021 was driven by COVID-19 deaths^28–33^, and excess deaths have generally exceeded reported COVID-19 deaths in both the USA and Europe^29,31,34^. In a study of 21 industrialized countries, including many European countries, excess deaths were 23% higher than reported COVID-19 deaths in early 2020^34^. However, the peaks and patterns in excess mortality have tracked closely with COVID-19 deaths in both the USA and Europe^35,36^.

Even though excess mortality is considered to assess the full impact of the pandemic^37^, other factors such as disruptions in health care access or utilization due to the pandemic may have contributed indirectly to excess deaths. Additionally, mortality may have declined in some groups or for some causes because of the pandemic, for example, decreases in mortality from influenza were observed during the pandemic^38^. It is unclear how these indirect effects affect excess mortality, and how those indirect effects of the pandemic vary between and within the USA and Europe.

This study has some limitations. For the USA, data from all 50 states and the District of Columbia were included, while for Europe, data were limited to the 25, mainly west-European, countries or subnational areas, and may not be representative of Europe as a whole. Other data sources of country-specific mortality data are available^2,19,39^ and it is unclear how results may differ when relying on alternative sources of mortality data. Estimates of excess mortality for the USA using the EuroMOMO method may differ somewhat from existing published estimates, though are within the range of previously published estimates from NCHS^29^. While existing estimates of excess mortality in the USA also typically rely on over-dispersed Poisson time series regression models^12^, differences in the algorithms used to estimate the expected numbers of deaths and the methods used to pool estimates across jurisdictions will cause results to vary. Other models or approaches to estimating excess mortality that account for factors such as population change or aging, or that include auxiliary explanatory variables such as infection rates, may result in different estimates^19,40–46^. The purpose of this study was to compare excess mortality patterns between the USA and Europe using a common methodology, and it is unlikely that using a different statistical model or approach would have resulted in substantially different patterns. While including explanatory variables related to viral surveillance metrics may have improved model fit, the availability and quality of these potential predictors varies by geography and time, introducing challenges with respect to potential differential measurement error and missingness. Further, weekly population estimates were not available, and were interpolated or extrapolated using available data, which may have affected the pattern in the estimated differences in excess mortality incidence rates. There have been substantial disparities in excess mortality by race/ethnicity throughout the pandemic^3,29,44,47–49^, and further evaluation of these inequities across different states, countries or regions would be valuable. While we adjusted for age, we did not adjust for sex, race/ethnicity, socioeconomic factors, or other potential covariates. In some cases, these variables may represent intermediates between country of residence and mortality, potentially explaining the differences between regions, which was beyond the scope of this descriptive analysis. Additionally, data availability and harmonization make that kind of detailed cross-country comparative analysis challenging.

In summary, through the COVID-19 pandemic from January 2020 to December 2021, both the USA and Europe experienced several waves of excess mortality. Over the two years, the USA had 44 more excess deaths per 100,000 person years than Europe. If the USA had experienced an excess mortality rate similar to the EuroMOMO countries or subnational areas, there would have been approximately 391 thousand (36%) fewer excess deaths in the USA. The age-specific differences in excess mortality varied, but excess mortality was consistently higher in the USA compared to Europe, except among children under 15, and among the older age groups at the start of the first wave in 2020 and the more recent wave at the end of 2021.

## Methods

### Data

#### USA

The US National Vital Statistics System provides weekly provisional counts of deaths from all causes occurring in the USA, by week of death, age group, and reporting jurisdiction (50 states, the District of Columbia [DC], and New York City)^12^. Data from 2015 through 2021 were used (based on data available as of the time of analysis in early April 2022). The provisional counts of deaths were weighted to account for incomplete reporting by jurisdictions in the most recent 26 weeks, where the weights were estimated based on completeness of provisional data in 2019. Population counts by jurisdiction and age group (as of July 1 in each year from 2015 to 2020) were obtained from the US Census Bureau^50^. Weekly population estimates were linearly interpolated between July 1 estimates each year. Estimates for the first half of 2015 were extrapolated backwards from the mid-year estimate, and 2021 estimates were extrapolated from 2020 as 2021 population estimates were not available.

#### Europe

Every week, 25 European countries or subnational areas (Austria, Belgium, Cyprus, Denmark, Estonia, England [United Kingdom], Finland, France, Germany, Greece, Hungary, Ireland, Italy, Luxembourg, Malta, the Netherlands, Northern Ireland [United Kingdom], Norway, Portugal, Scotland [United Kingdom], Slovenia, Spain, Sweden, Switzerland and Wales [United Kingdom]) adjust their data for delay in registration (primarily affecting the most recent 6 weeks) and run local analyses of excess deaths from all causes^13^. Aggregated data by week and age group are sent to the EuroMOMO hub at Statens Serum Institut, Denmark. At the hub, data are pooled^11^ to provide European estimates of weekly expected numbers of deaths and excess numbers of deaths, from all causes^13^. Data from 2015 through December 31, 2021 were used.

Population sizes, observed (≤ 2020) and forecasted (2021), for each of the countries or subnational areas (as of January 1 in each year from 2015–2021) were downloaded from EuroSTAT^51^. Weekly population estimates were linearly interpolated from the yearly January 1 data. For three countries reporting weekly numbers of deaths for a subpopulation (Italy, France, and Spain)^52^, their respective populations were adjusted proportionally.

#### Patient and public involvement

No patients were involved in this study. All methods were carried out in accordance with relevant guidelines and regulations. This was a register-based study relying on deidentified, aggregated, publicly available secondary data and an ethical approval is not needed.

### Statistical analyses

#### The EuroMOMO model

The EuroMOMO statistical model fits over-dispersed Poisson time series (with weekly data based on International Organization for Standardization [ISO] weeks) regression models to predict the expected number of deaths^11,14^. Models include terms for annual trends and seasonality (sine curve), and a post-estimation 2/3-power transformation correction for skewness in the predictive distribution^14^. The baseline expected numbers are estimated based on predefined weeks in spring and autumn, when excess mortality is assumed to be ignorable under normal conditions, using data from the five preceding years (and the present season/year) to ensure stable estimates.

Estimates from stratified analyses of individual countries or subnational areas were pooled, thus accounting for heterogeneity between countries^11^. Similarly, estimates from individual reporting jurisdictions in the USA were pooled, to account for between-jurisdiction heterogeneity. In this pooling process, stratified analyses are conducted to estimate baseline expected numbers of deaths, uncertainty intervals, and numbers of excess deaths for each individual country or jurisdiction separately. These estimates are then summed, and corresponding variance estimates are pooled across the larger geographic regions (i.e., Europe and the USA) using formulas available in Nielsen et al.^11^.

During the pandemic, the assumption about ignorable excess mortality during spring and autumn weeks was violated, so data for the weeks during the pandemic were excluded from the estimation of the baseline expected deaths. The baseline expected deaths were based solely on data from the five years preceding the pandemic, 2015–2019.

#### Mortality incidence rates

To account for differences in population sizes between the USA and Europe, incidence rates per 100,000 person years were calculated for the expected baseline numbers of deaths, observed numbers of deaths and excess numbers of deaths.

#### Comparisons

Mortality incidence rates were age-standardized using the direct method based on the joint weekly age distribution between the USA and the EuroMOMO countries or subnational areas throughout the study period, to account for differences in the age structure of the two populations. Rate differences in excess mortality incidence rates between the USA and Europe were calculated by subtracting rates for Europe from rates for the USA. Comparisons were made across the whole period (2020-W01 to 2021-W52) and for three time periods reflecting the three primary waves in excess mortality (wave 1: 2020-W01 to 2020-W39, wave 2: 2020-W40 to 2021-W26 and wave 3: 2021-W27 to 2021-W52).

Estimates of uncertainty around the rates or numbers of deaths are shown as 95% uncertainty intervals (± 1.96 standard deviations).

Pooled weekly data for the USA and Europe for 2020 and 2021 are available on https://github.com/JensXII/USA_EuroMOMO_ 2020_2021.

All analyses were done using the statistical software R version 4.1.3 (R Foundation for Statistical Computing, https://www.R-project.org/).

### Ethics approval

This a register-based study relying on deidentified, aggregated publicly available data and an ethical approval is not needed.

## Supplementary Information

Supplementary Information.

## Data Availability

The data sets analyzed during the current study are available in the GitHub repository https://github.com/JensXII/USA_EuroMOMO_2020_2021.
