# Supplementary material for: Excess all-cause mortality in the USA and Europe during the COVID-19 pandemic, 2020 and 2021
Source: Sci Rep. 2022 Nov 3;12:18559. doi: 10.1038/s41598-022-21844-7 (PMC9630804; doi:10.1038/s41598-022-21844-7)

**Supplementary Information**

**Excess all-cause mortality in the USA and Europe during the COVID-19 pandemic, 2020 and 2021**

Lauren M. Rossen, PhD, Sarah K Nørgaard, PhD, Paul D. Sutton, PhD, Tyra G Krause, PhD, Farida B. Ahmad, MPH, Lasse S. Vestergaard, PhD, Kåre Mølbak, DMSc, Robert N. Anderson, PhD, Jens Nielsen, PhD

**Supplementary Figure S1:** Pooled weekly excess mortality rates (excess deaths per 100,000 person years), from 2020-2021 in the USA: ages 0-14 years


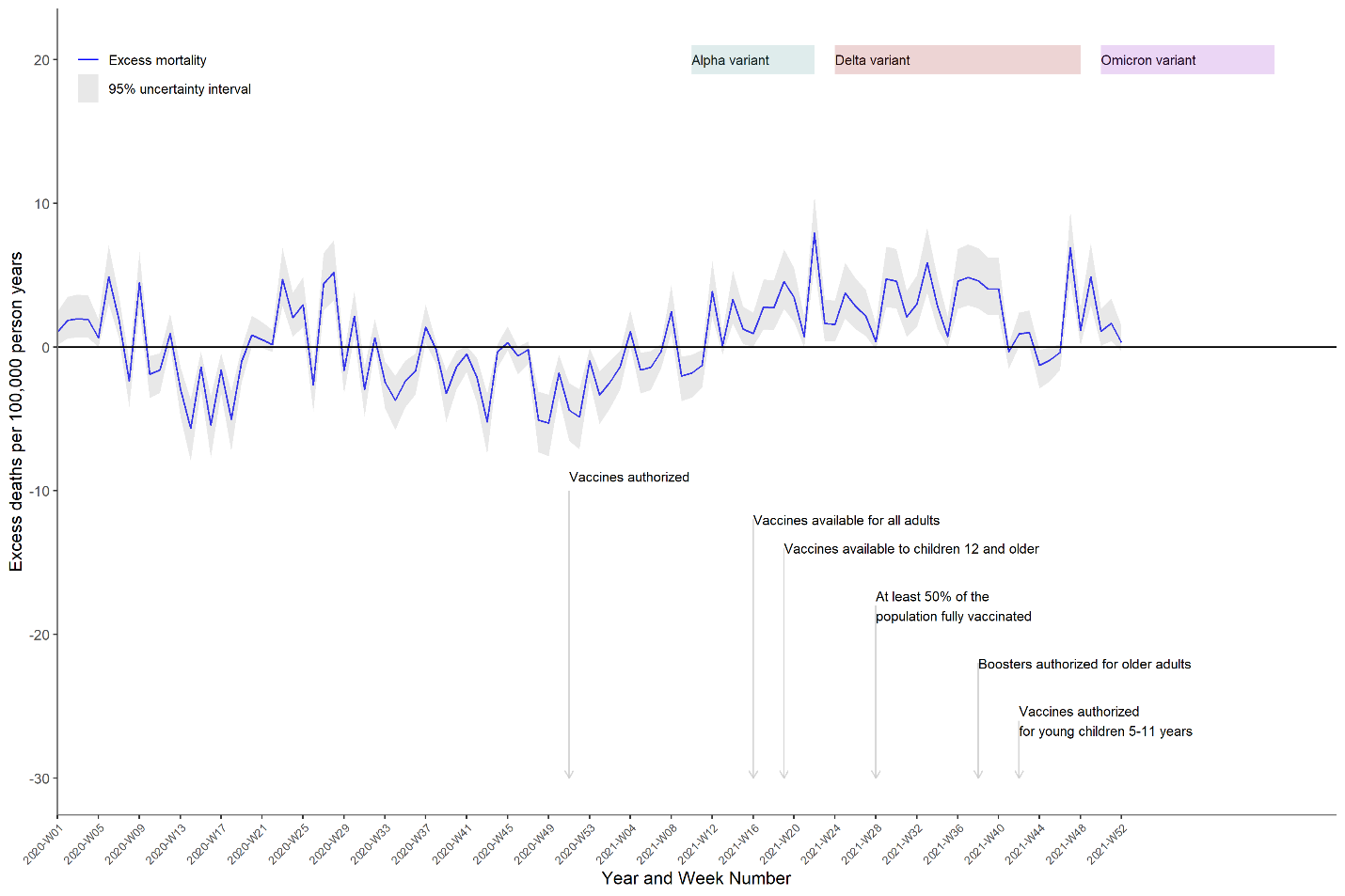


**Note:** Dates related to vaccine milestones and predominant variants are approximate, as there was substantial variation across jurisdictions within the USA as to when some of these milestones occurred.

**Supplementary Figure S2:** Pooled weekly excess mortality rates (excess deaths per 100,000 person years), from 2020-2021 in the USA: ages 15-44 years


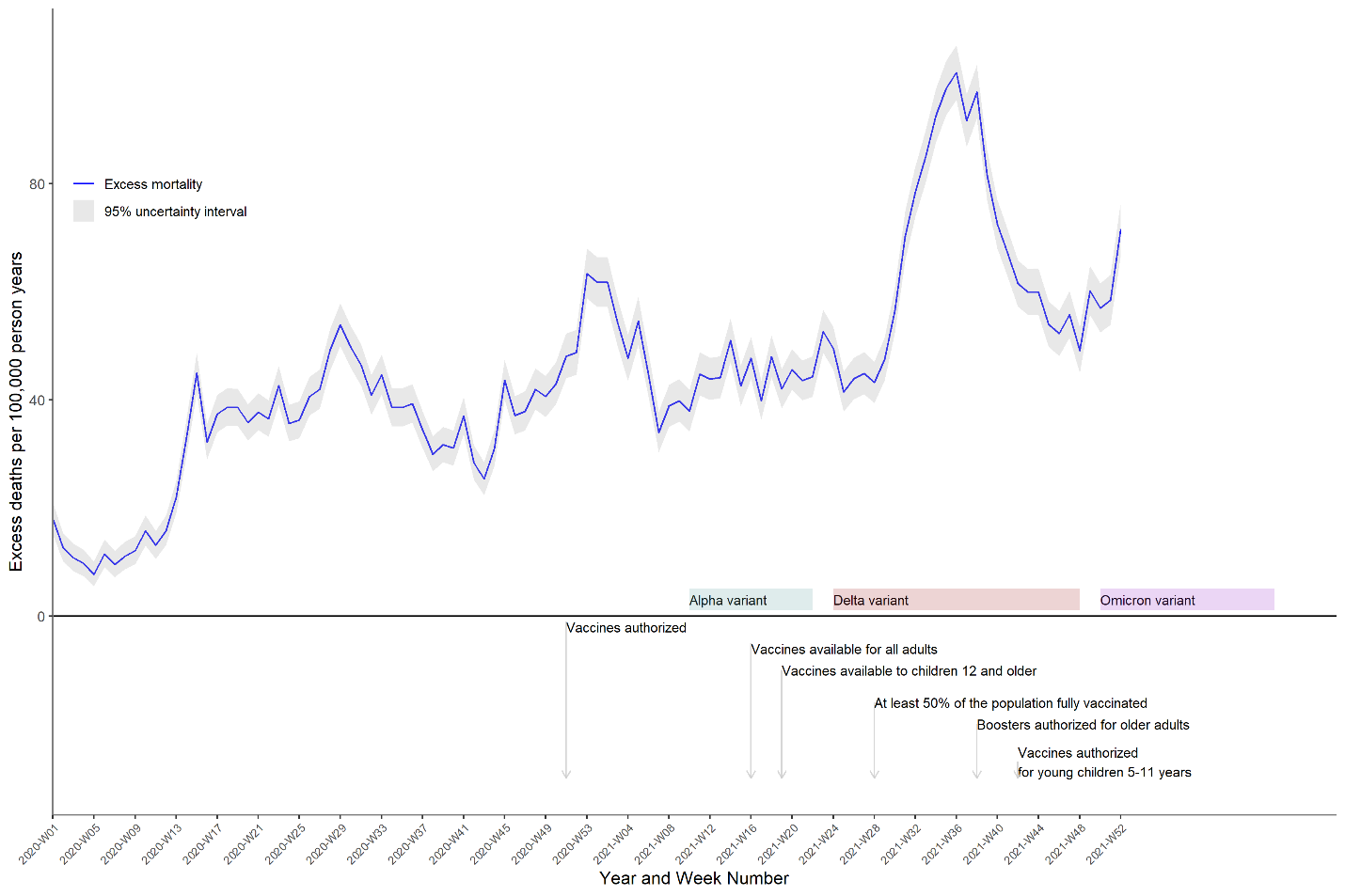


**Note:** Dates related to vaccine milestones and predominant variants are approximate, as there was substantial variation across jurisdictions within the USA as to when some of these milestones occurred.

**Supplementary Figure S3:** Pooled weekly excess mortality rates (excess deaths per 100,000 person years), from 2020-2021 in the USA: ages 45-64 years


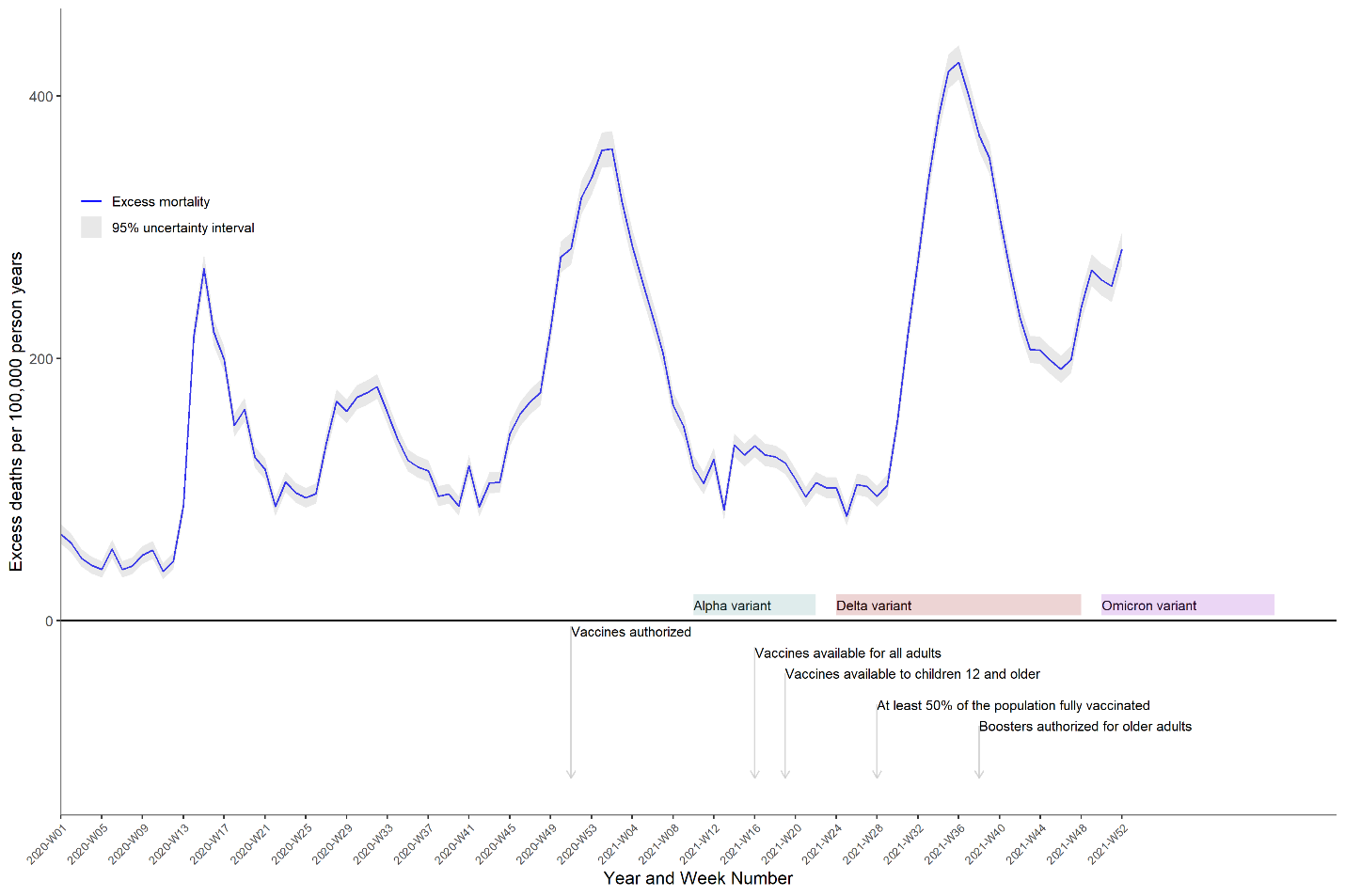


**Note:** Dates related to vaccine milestones and predominant variants are approximate, as there was substantial variation across jurisdictions within the USA as to when some of these milestones occurred.

**Supplementary Figure S4:** Pooled weekly excess mortality rates (excess deaths per 100,000 person years), from 2020-2021 in the USA: ages 65-74 years


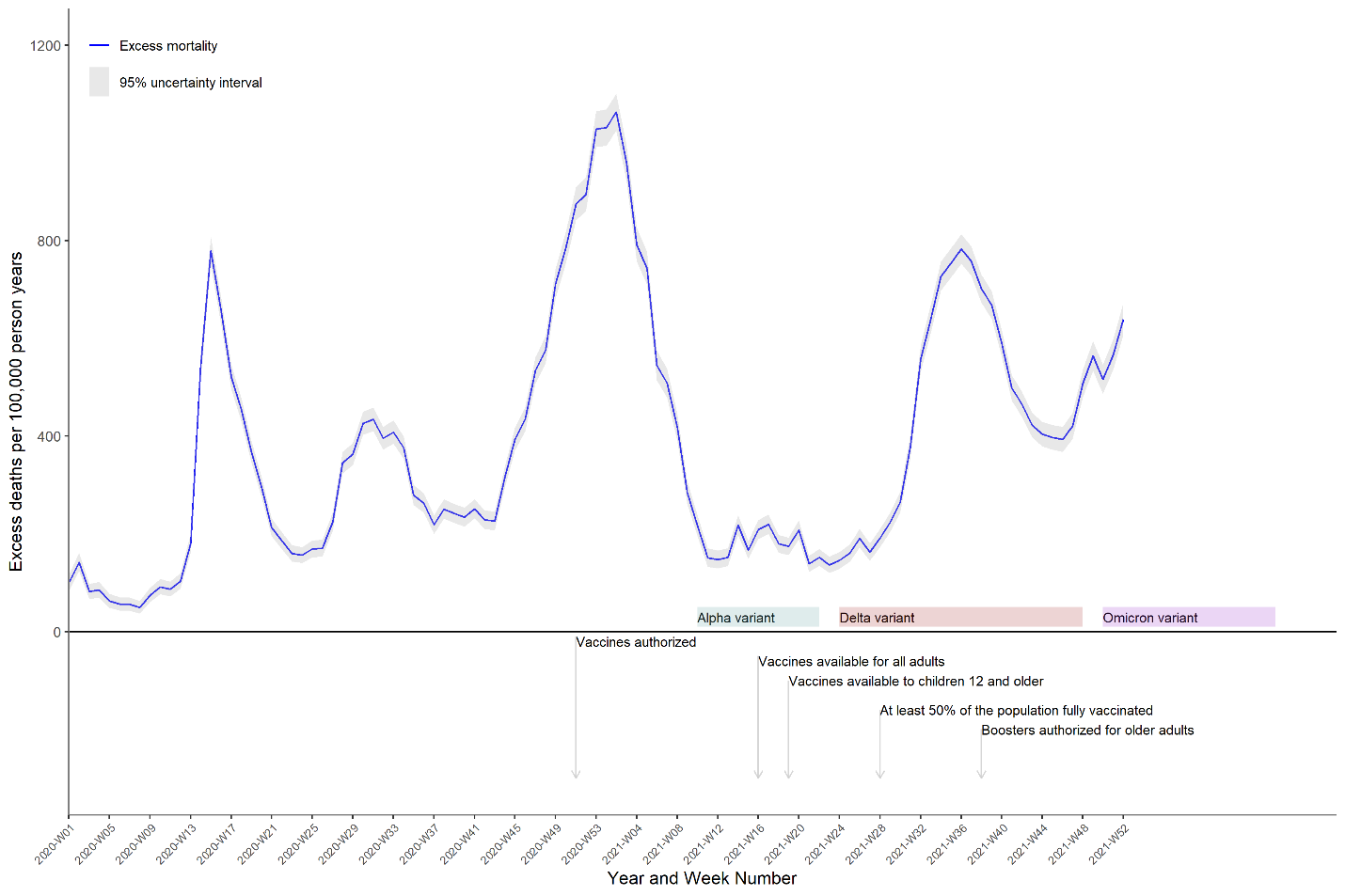


**Note:** Dates related to vaccine milestones and predominant variants are approximate, as there was substantial variation across jurisdictions within the USA as to when some of these milestones occurred.

**Supplementary Figure S5:** Pooled weekly excess mortality rates (excess deaths per 100,000 person years), from 2020-2021 in the USA: ages 75-84 years


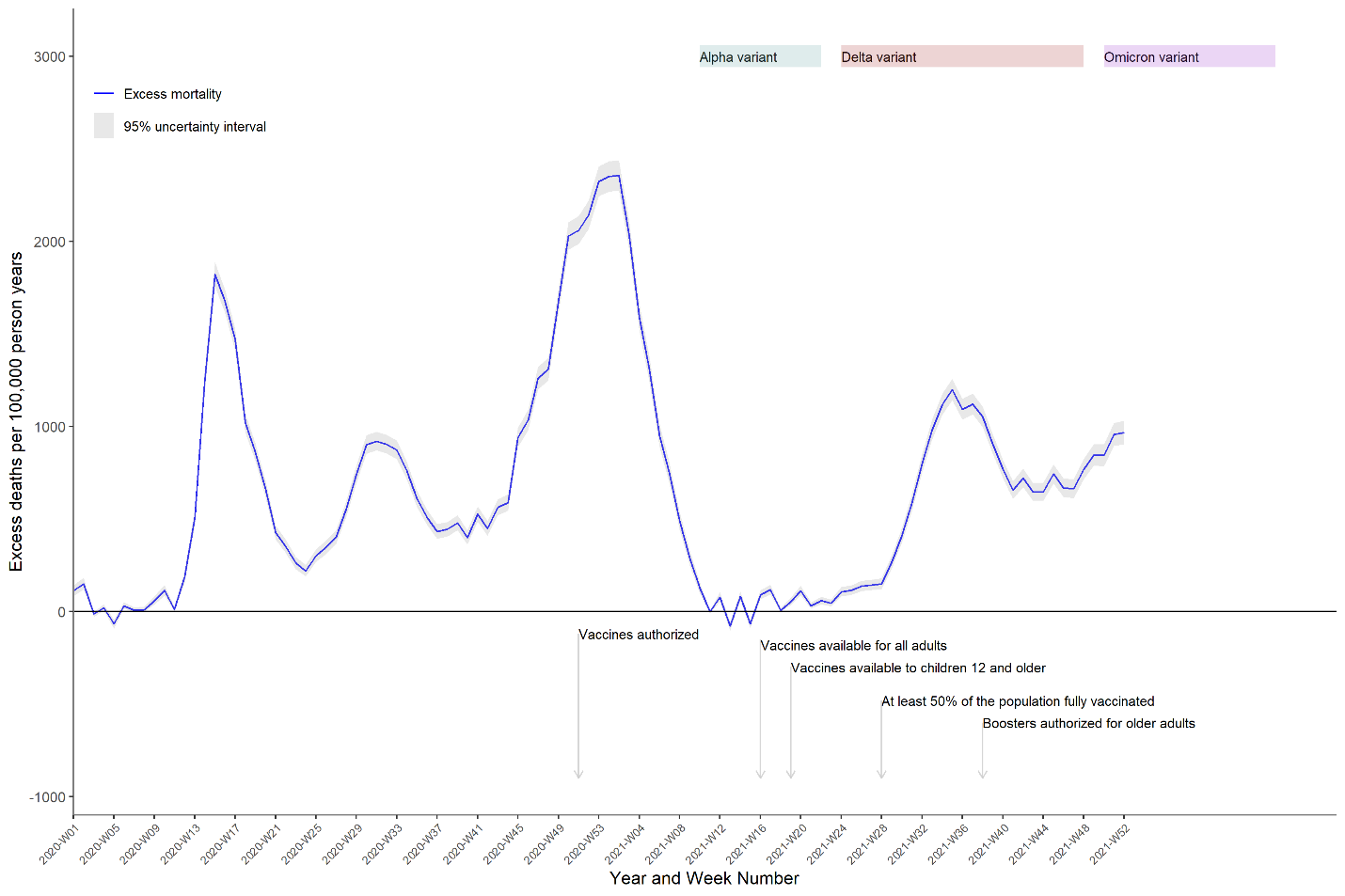


**Note:** Dates related to vaccine milestones and predominant variants are approximate, as there was substantial variation across jurisdictions within the USA as to when some of these milestones occurred.

**Supplementary Figure S6:** Pooled weekly excess mortality rates (excess deaths per 100,000 person years), from 2020-2021 in the USA: ages 85 years and older


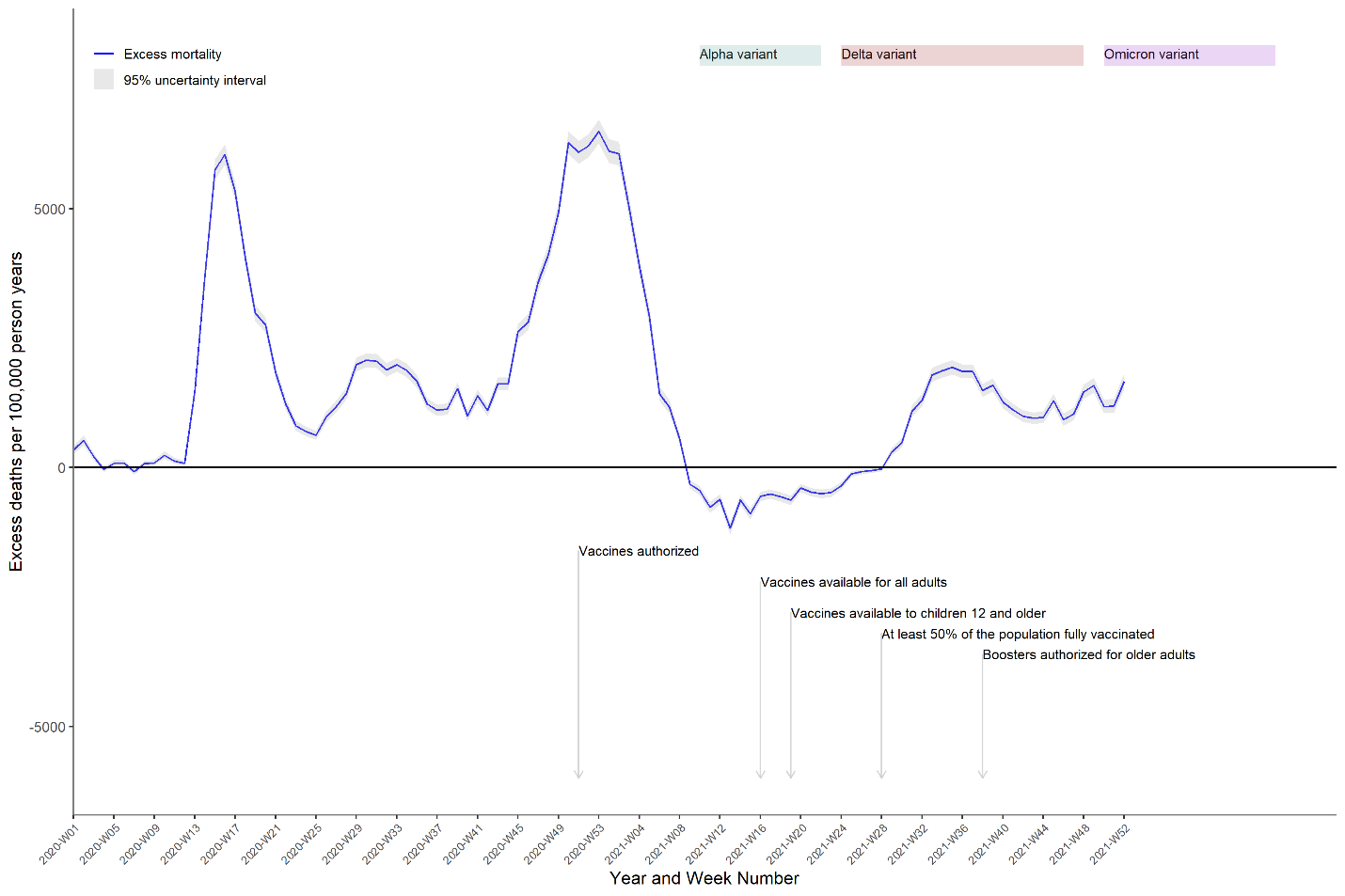


**Note:** Dates related to vaccine milestones and predominant variants are approximate, as there was substantial variation across jurisdictions within the USA as to when some of these milestones occurred.

**Supplementary Figure S7:** Pooled weekly excess mortality rates (excess deaths per 100,000 person years), from 2020-2021 in the USA: all ages


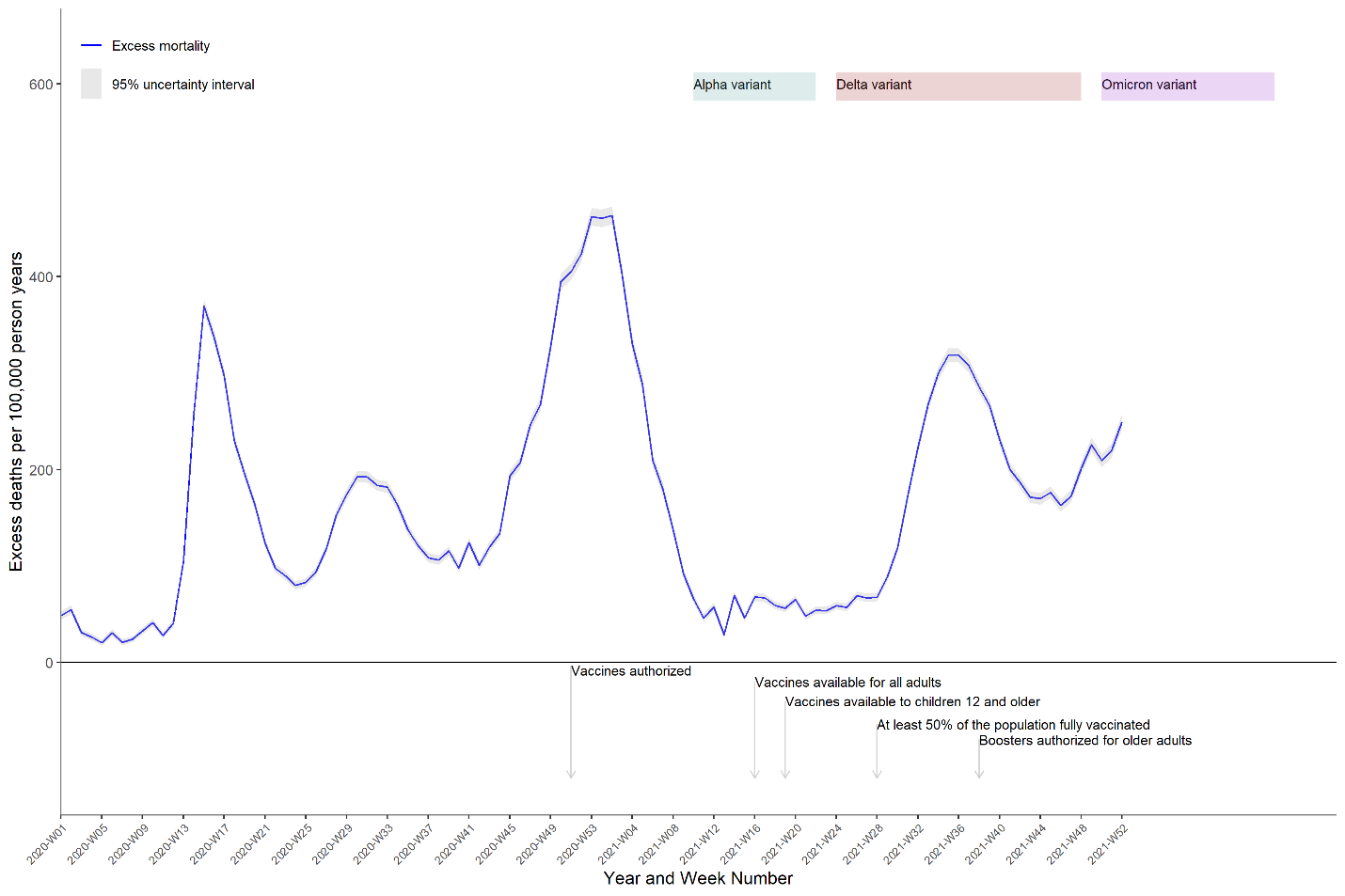


**Note:** Dates related to vaccine milestones and predominant variants are approximate, as there was substantial variation across jurisdictions within the USA as to when some of these milestones occurred.

**Supplementary Figure S8:** Pooled weekly excess mortality rates (excess deaths per 100,000 person years), from 2020-2021 in Europe: ages 0-14 years


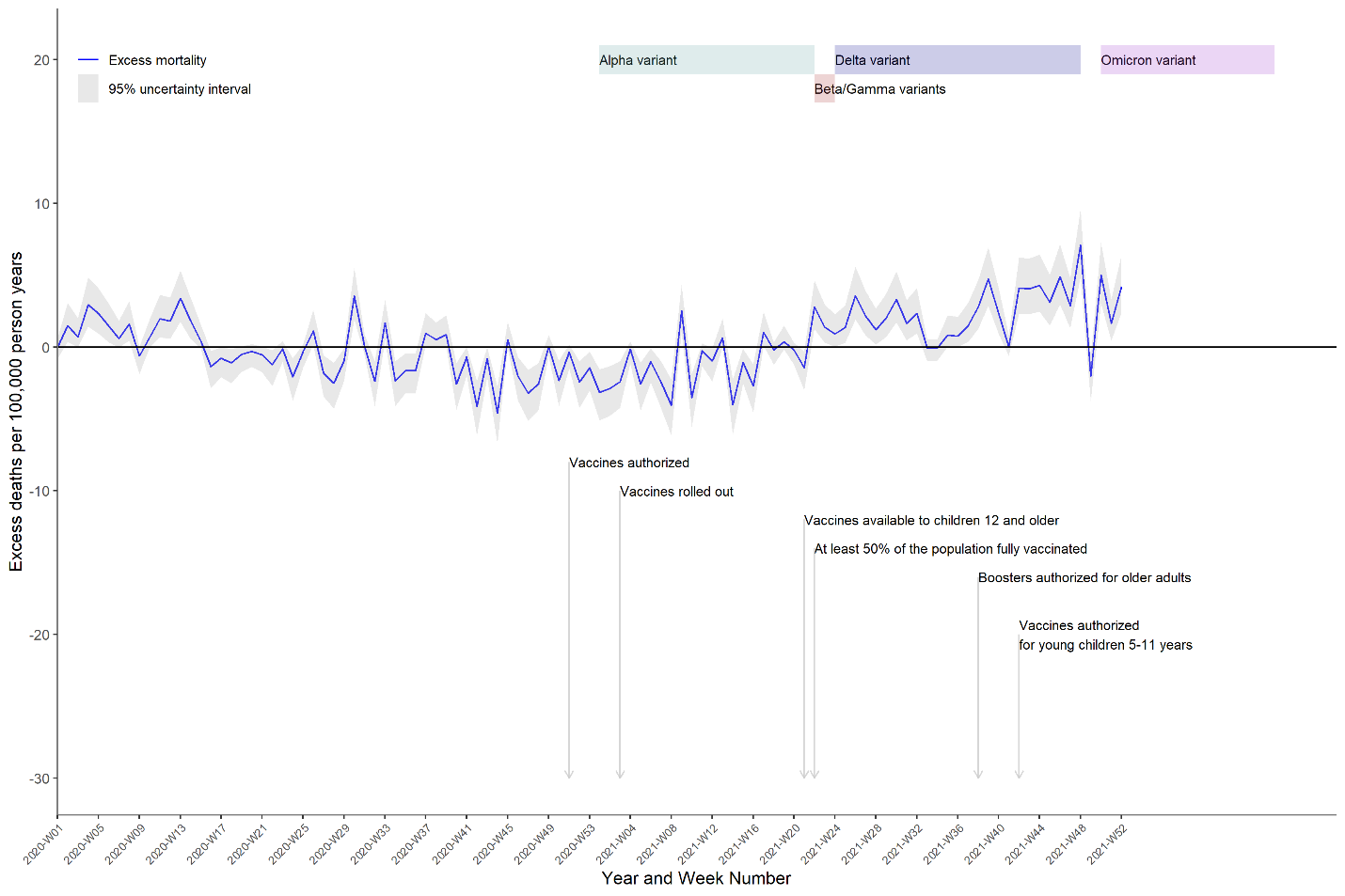


**Note:** Dates related to vaccine milestones and predominant variants are approximate, as there was substantial variation across European countries as to when some of these milestones occurred.

**Supplementary Figure S9:** Pooled weekly excess mortality rates (excess deaths per 100,000 person years), from 2020-2021 in Europe: ages 15-44 years


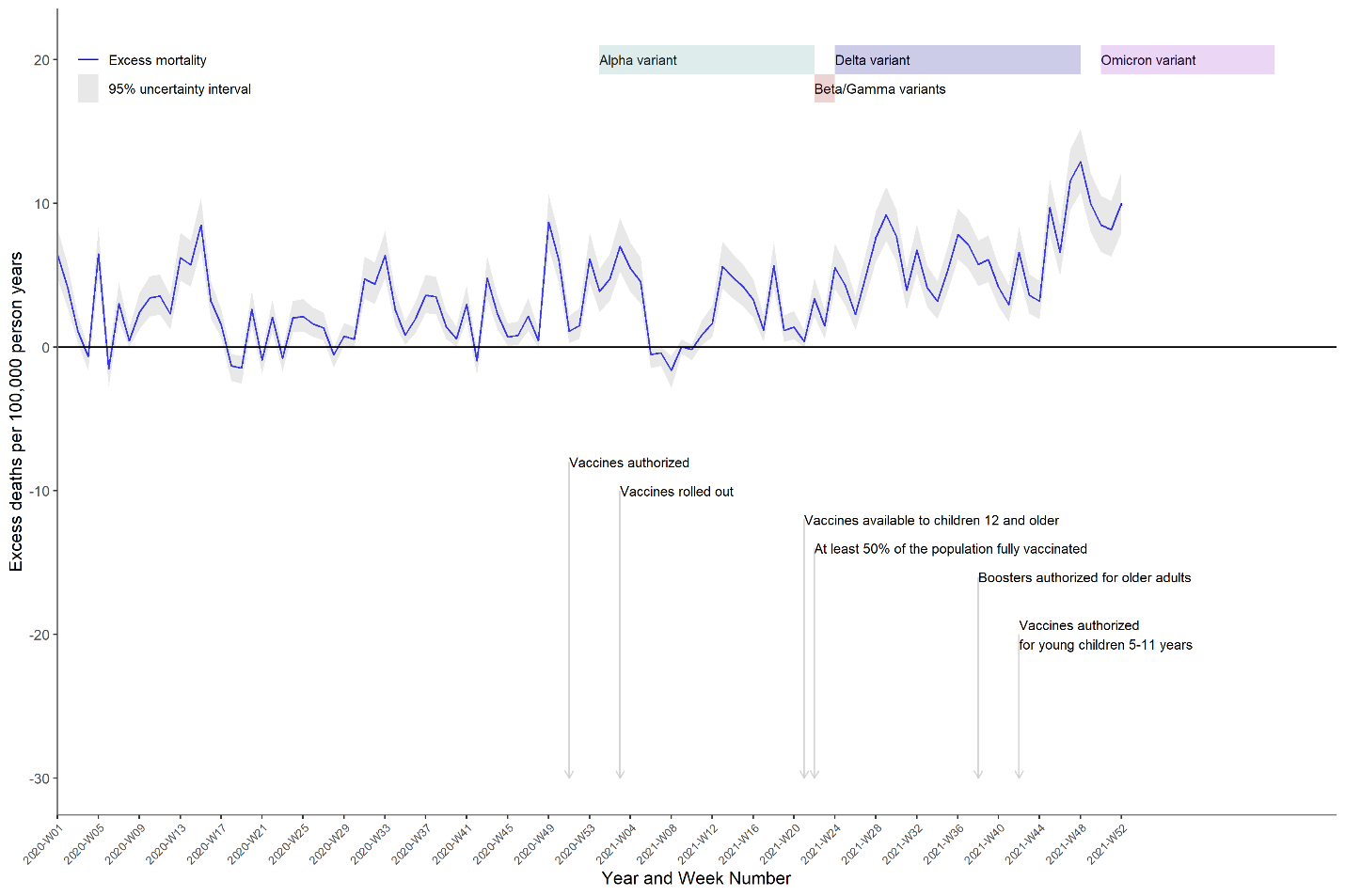


**Note:** Dates related to vaccine milestones and predominant variants are approximate, as there was substantial variation across European countries as to when some of these milestones occurred.

**Supplementary Figure S10:** Pooled weekly excess mortality rates (excess deaths per 100,000 person years), from 2020-2021 in Europe: ages 45-64 years


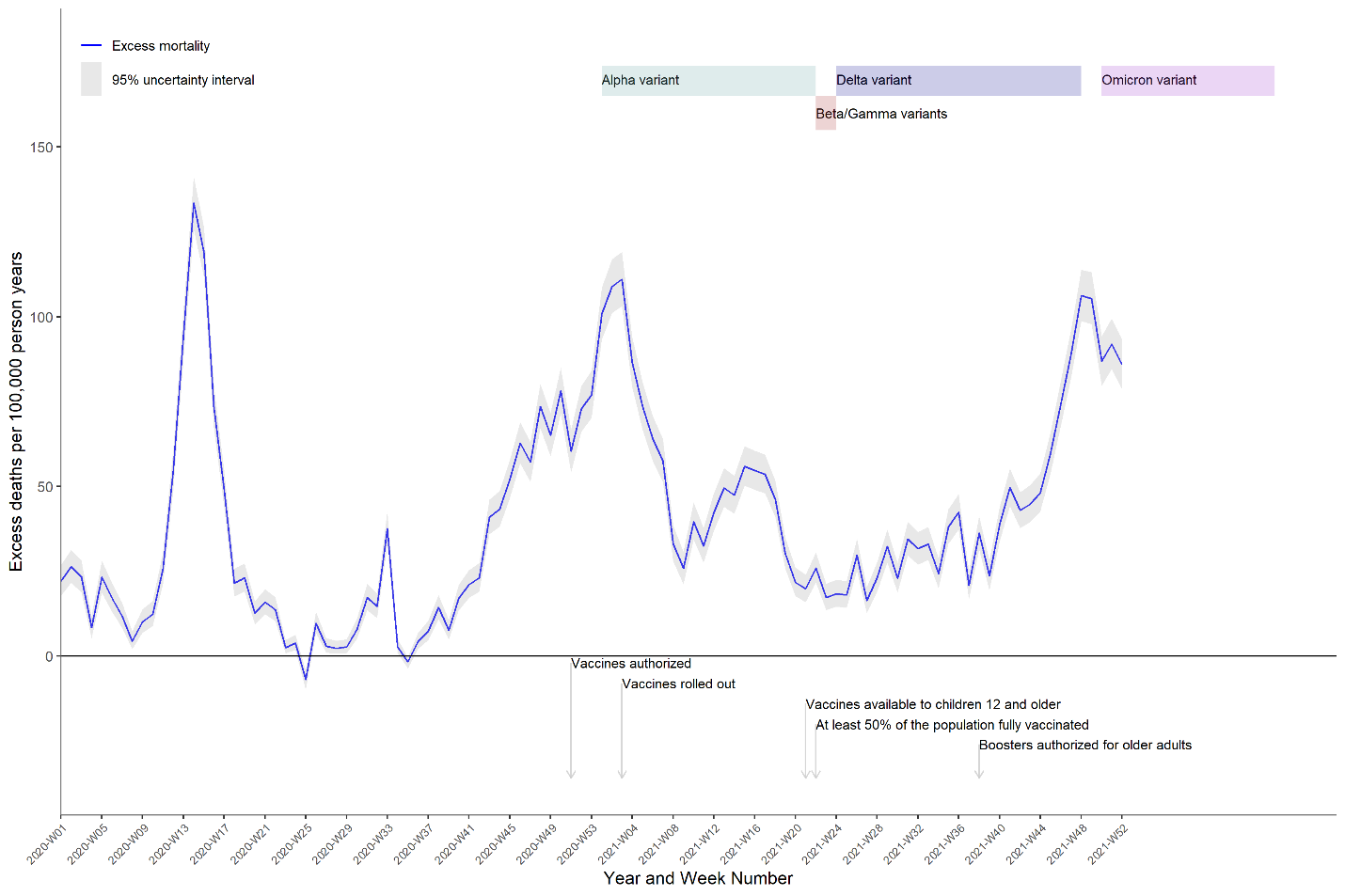


**Note:** Dates related to vaccine milestones and predominant variants are approximate, as there was substantial variation across European countries as to when some of these milestones occurred.

**Supplementary Figure S11:** Pooled weekly excess mortality rates (excess deaths per 100,000 person years), from 2020-2021 in Europe: ages 65-74 years


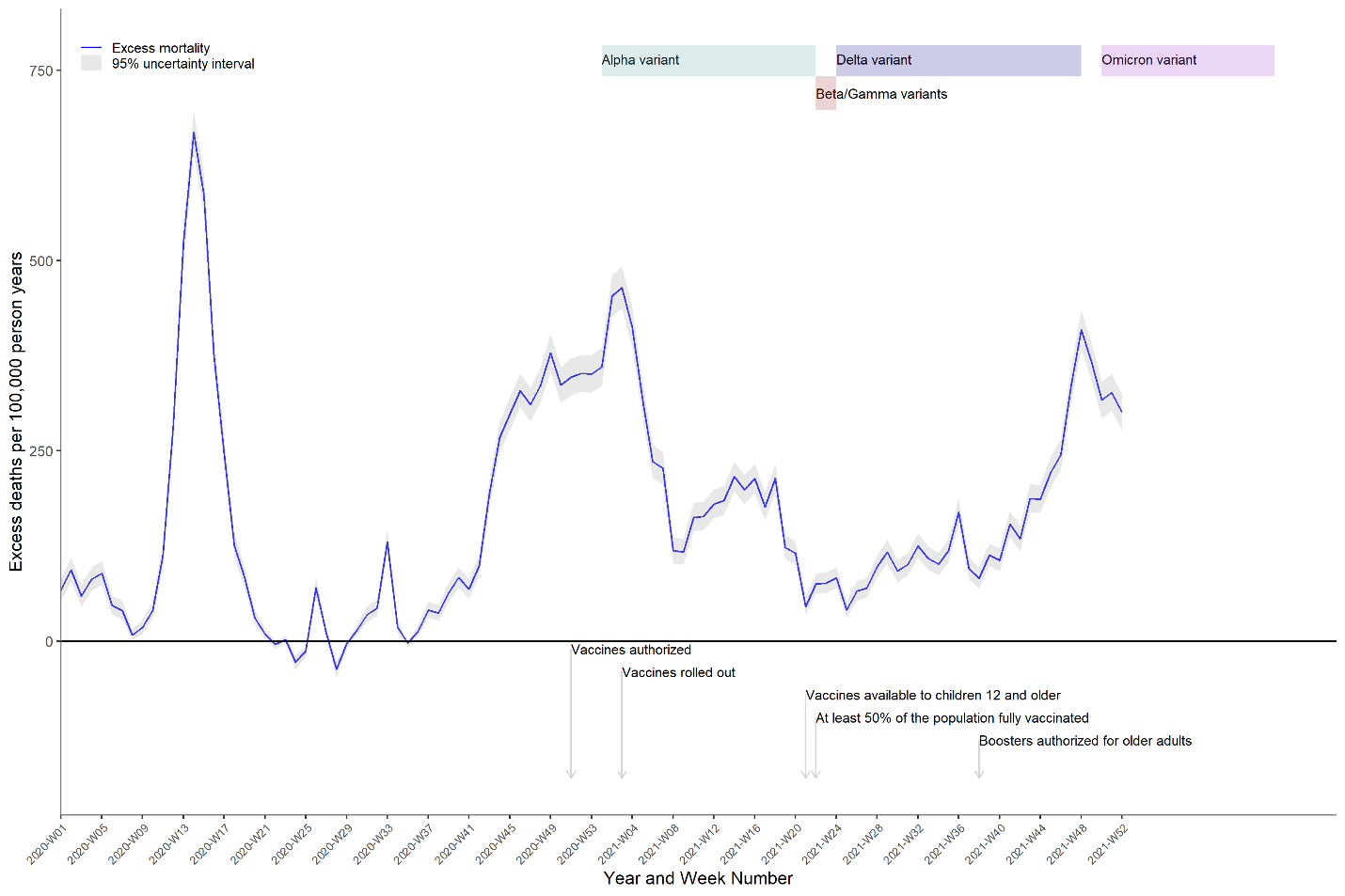


**Note:** Dates related to vaccine milestones and predominant variants are approximate, as there was substantial variation across European countries as to when some of these milestones occurred.

**Supplementary Figure S12:** Pooled weekly excess mortality rates (excess deaths per 100,000 person years), from 2020-2021 in Europe: ages 75-84 years


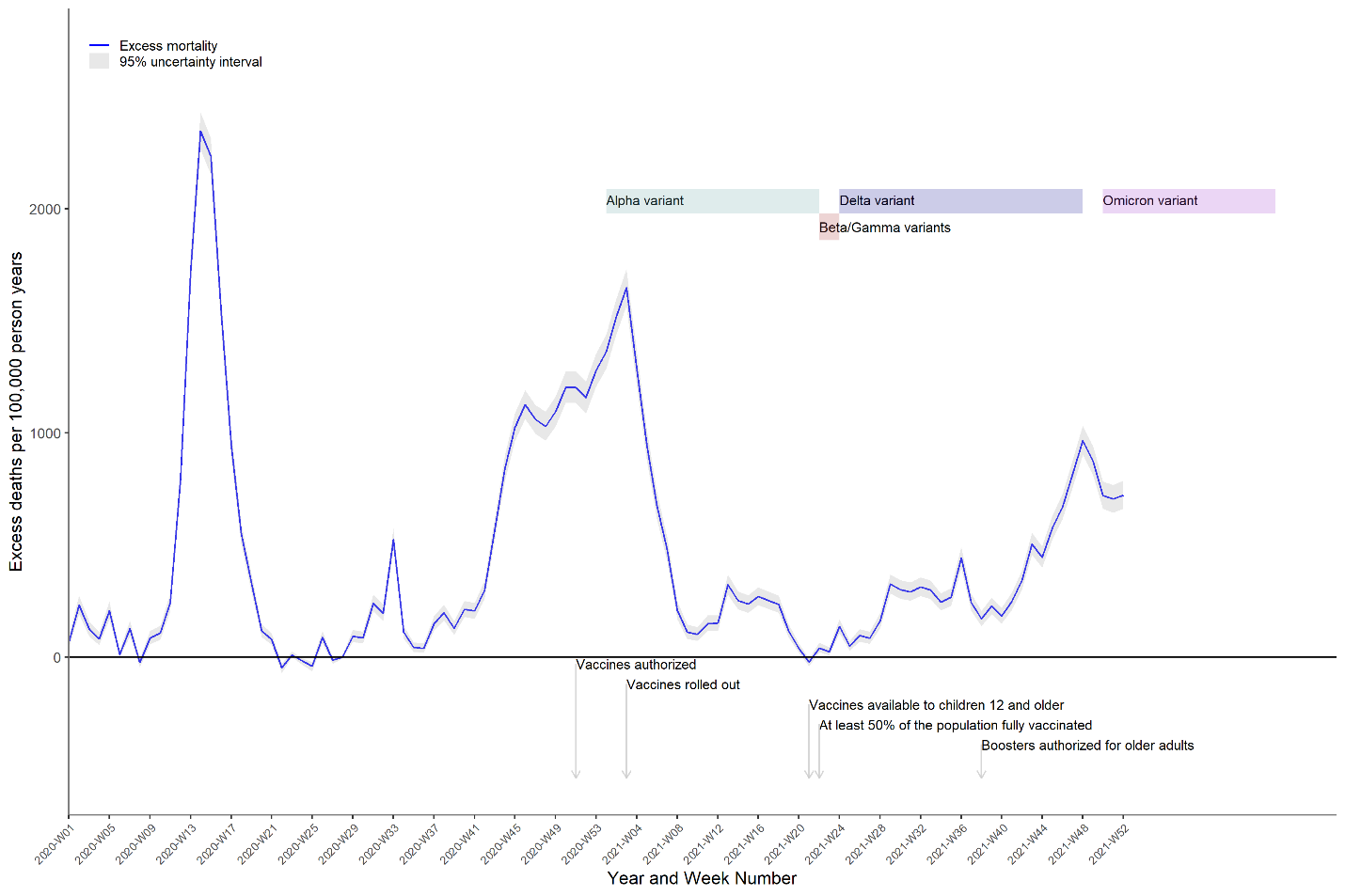


**Note:** Dates related to vaccine milestones and predominant variants are approximate, as there was substantial variation across European countries as to when some of these milestones occurred.

**Supplementary Figure S13:** Pooled weekly excess mortality rates (excess deaths per 100,000 person years), from 2020-2021 in Europe: ages 85 years and older


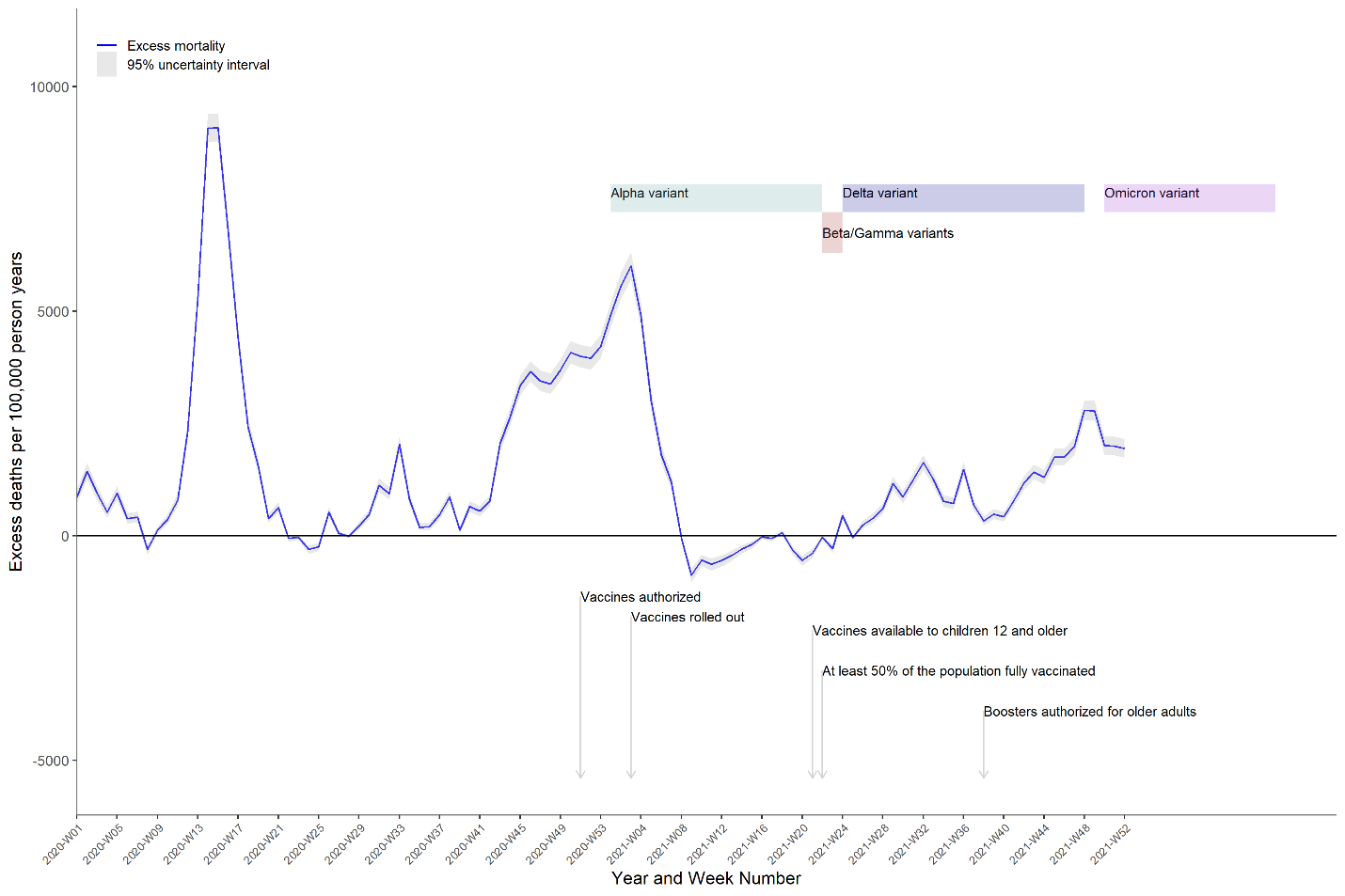


**Note:** Dates related to vaccine milestones and predominant variants are approximate, as there was substantial variation across European countries as to when some of these milestones occurred.

**Supplementary Figure S14:** Pooled weekly excess mortality rates (excess deaths per 100,000 person years), from 2020-2021 in Europe: all ages


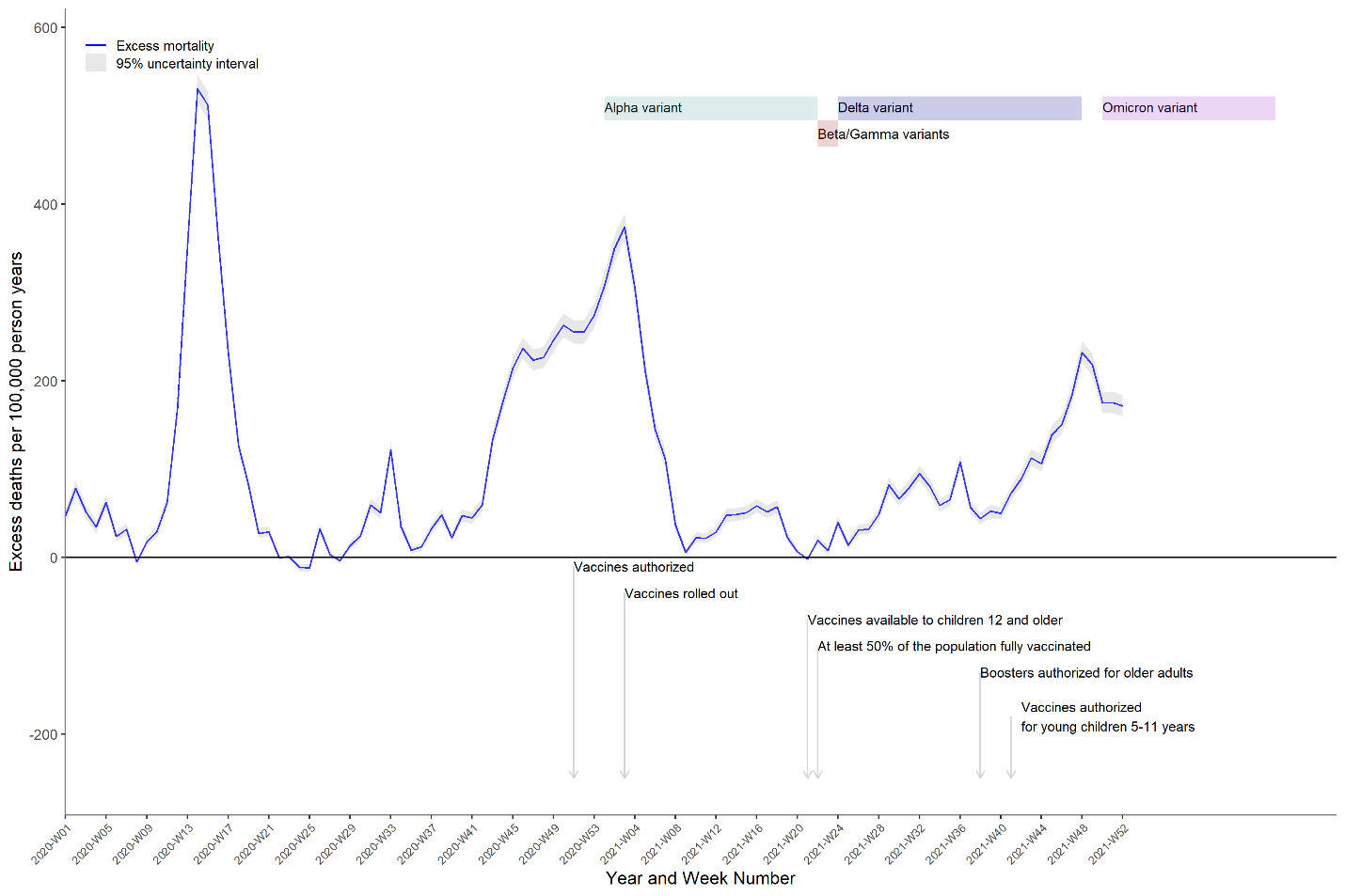


**Note:** Dates related to vaccine milestones and predominant variants are approximate, as there was substantial variation across European countries as to when some of these milestones occurred.

**Supplementary Figure S15:** Pooled weekly expected and observed all-cause mortality rates (deaths per 100,000 person years), from 2017-2021: USA and Europe.


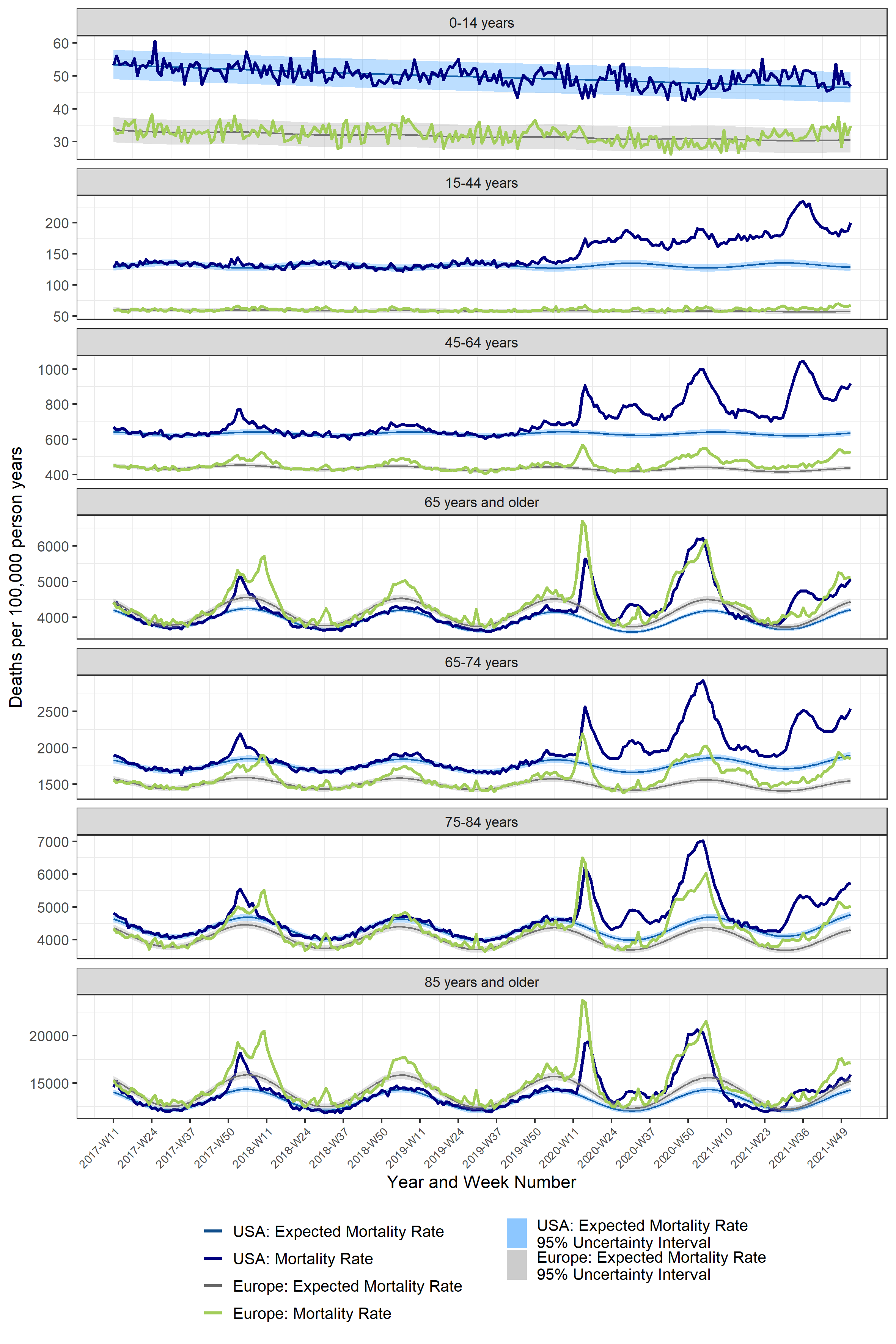

Supplement: Supplementary file 1 — Supplementary Information. [file 41598_2022_21844_MOESM1_ESM.docx]
